# Cervical invasion, lymphovascular space invasion, and ovarian metastasis as predictors of lymph node metastasis and poor outcome on stages I to III endometrial cancers: a single-center retrospective study

**DOI:** 10.1186/s12957-019-1733-2

**Published:** 2019-11-16

**Authors:** Min Li, Shuwei Wu, Yangqin Xie, Xiaohui Zhang, Zhanyu Wang, Ying Zhu, Shijie Yan

**Affiliations:** Department of Obstetrics and Gynecology, the First Affiliated Hospital of Anhui Medical University, 218 Jixi Road, Hefei, 230022 Anhui China

**Keywords:** Endometrial cancer, Endometrial carcinoma, Lymph node metastasis, Lymph node dissection, Lymphadenectomy

## Abstract

**Background:**

The aim of this study is to determine pathological factors that increase the risk of LNM and indicate poor survival of patients diagnosed with endometrial cancer and treated with surgical staging.

**Method:**

Between January 2010 and November 2018, we enrolled 874 eligible patients who received staging surgery in the First Affiliated Hospital of Anhui Medical University. The roles of prognostic risk factors, such as age, histological subtype, tumor grade, myometrial infiltration, tumor diameter, cervical infiltration, lymphopoiesis space invasion (LVSI), CA125, and ascites, were evaluated. Multivariable logistic regression models were used to identify the predictors of LNM. Kaplan–Meier and COX regression models were utilized to study the overall survival.

**Results:**

Multivariable regression analysis confirmed cervical stromal invasion (OR 3.412, 95% CI 1.631–7.141; *P* < 0.01), LVSI (OR 2.542, 95% CI 1.061–6.004; *P* = 0.04) and ovarian metastasis (OR 6.236, 95% CI 1.561–24.904; *P* = 0.01) as significant predictors of nodal dissemination. Furthermore, pathological pattern (*P* = 0.03), myometrial invasion (OR 2.70, 95% CI 1.139–6.40; *P* = 0.01), and lymph node metastasis (OR 9.675, 95% CI 3.708–25.245; *P* < 0.01) were independent predictors of decreased overall survival.

**Conclusions:**

Cervical invasion, lymphopoiesis space invasion, and ovarian metastasis significantly convey the risk of LNM. Pathological type, myometrial invasion, and lymph node metastasis are all important predictors of survival and should be scheduled for completion when possible in the surgical staging procedure.

## Background

Endometrial cancer (EC), the most frequent form of female genital tract tumors [1], lead to approximately 20% mortality worldwide. Recently, the incidence of endometrial cancer has shown an upward trend year by year [2]. Lymph node metastasis (LNM) is widely recognized as the main prognostic factor for endometrial cancer [3].

So far, routine procedures for EC staging and treatment are total hysterectomy and bilateral salpingo-oophorectomy, including pelvic and para-aortic lymphadenectomy [4]. However, whether lymph node dissection (LND) could be applied for endometrial cancer patients remains a controversial issue, either in stage I or in a higher stage [5, 6]. Many debates about the role of lymphadenectomy exist. According to National Comprehensive Cancer Network (NCCN), surgical staging could be performed in EC patients, and referred to for adjuvant therapy [7]. Wang et al. consider patients with early stage disease should take surgical staging [8]. While some contemporary trials take exception to the effects of lymphadenectomy since it does not prolong overall and disease-free survival time [9, 10]. Therefore, it is necessary to recognize patients who will profit from lymphadenectomy.

One of the challenges in EC is to determine the specific factors that predict lymph node metastasis, so as to optimize the patient group receiving LA and estimate the need for adjuvant therapy. Due to good prognosis of primary endometrial cancer, it is possible and necessary to avoid over treatment, especially for elderly women on whom often increases the risk of complications [11]. Lymphoedema is a well-known long-term complication following LA, and its incidence may increase over time [12]. Much work so far has focused on the relationship between pathological characteristics and lymph node metastasis. There is still no consensus. Therefore, further studies are still required.

In this study, we aim to retrospectively identify pathological factors that increase the risk of LNM and indicate poor survival in patients diagnosed with stages I–III endometrial cancer and treated with surgical staging.

## Methods

Using a retrospective database at the Department of Obstetrics and Gynecology, First Affiliated Hospital of Anhui Medical University, all endometrial cancer patients receiving complete staging surgery between January 2010 and November 2018 (*n* = 874) were enrolled. The study program was approved by reviewing committee of provincial institutions.

The study population included women with endometrial cancer clinically confined to the uterus who underwent comprehensive surgical staging. Women with high risk factors for nodal metastasis were excluded. These risk factors were as follows: FIGO (International Federation of Gynecology and Obstetrics, 2009) stage IV disease, presence of synchronous carcinoma, experiences of prior neoadjuvant chemotherapy or radiotherapy, along with a secondary malignity. Patients with incomplete data were also excluded from the study. All eligible candidates were divided into “group 1” patients without LNM and “group 2” patients with positive nodes.

Relationship between clinical parameters and lymph node metastasis or overall survival was assessed. Clinical data and tumor characteristics, including age at surgery, histological subtype, tumor grade, depth of myometrial invasion, cervical stromal invasion, peritoneal cytology, primary tumor size (PTD), lymphatic vascular infiltration, and preoperative CA125 level, were extracted from patients’ medical records and original pathology reports.

Surgical staging includes total hysterectomy, bilateral salpingectomy, pelvic and aortic lymphadenectomy, and peritoneal irrigation. All patients entering into the study received comprehensive surgical staging—365 (83.5%) patients with laparotomy while 72 (16.5%) cases of laparoscopy surgery. Pelvic lymph node resection refers to the removal of all lymph nodes, including common ilium lymph nodes, internal ilium lymph nodes, external ilium lymph nodes, and obturator lymph nodes. For grade 3 tumors, paraortic lymph node dissection was performed on high risk patients, those with deep myometrium invasion(> 50%), and cervical involvement. Resection of paraortic lymph node involves lymph nodes located at the level of the aortic bifurcation to the renal vein, with the area above the (IMA) of the submesenteric artery contained [13]. All surgical procedures were conducted by gynecological oncologists. The operative data included the total number of LNs resection, the number of pelvic LNs resection, and the number of LNs removal subsequent to the aorta.

All surgical specimens were reviewed and explained by gynecological pathologists. Depending on the World Health Organization classification, the histology classification was carried out [14]. The primary tumor size (PTD), defined as the largest diameter in each of the three dimensions of the tumor, was measured on fresh tissue by pathologists [15]. The overall survival was considered as the time from primary surgery to death or the last follow-up [16]. All tumors were staged according to the FIGO staging system (2009) [17].

The systematic lymph node dissection (LND) is defined as excision of more than 20 nodes [18]. Appropriate pelvic lymphadenectomy is defined as the removal of at least ten pelvic lymph nodes, and appropriate para-aortic lymphadenectomy is defined as the removal of at least five para-aortic lymph nodes [19, 20]. Adjuvant therapies are at the discretion of the therapist, including chemotherapy, radiotherapy, hormone therapy, or some combination of these treatments.

Statistical analysis was performed using Statistical Package Social Sciences (SPSS)23.0. Continuous variables (presented as mean ± SD or median (range)) were analyzed using the Kolmogorov–Smirnov test to determine whether they were normally distributed. Then Mann–Whitney *U* test was utilized for non-normal data. As to categorical variable (presented as number and percentage), the chi-square test (Pearson chi-square and Pearson exact chi-square tests) was used to compare the proportions between groups. Univariate and multivariate logistic models were utilized to recognize the risk factors. The Kaplan–Meier method and cox regression were used to evaluate the covariance and generate the survival curves, whose comparisons were made with the log rank test and Breslow (Generalized Wilcoxon) test. A *P* value < 0.05 was identified as statistically significant.

## Results

A total of 874 patients met the inclusion criteria of this research and were included. According to the final pathology reports, lymph nodes involvement was found in 82 (9.61%) patients. Of the 874 patients, 570 (65.22%) presented with FIGO stage I, 212 (24.26%) with stage II, and 92 (10.52%) with stage III disease. There were 792 in group 1 and 82 in group 2. Baseline clinicopathological characteristics of the cohort were all provided in Table 1. The average age was 53.58 years for all subjects, 53.74 and 51.79 years for group 1 and group 2, respectively. The intermediate number of pelvic lymph nodes harvested was 13 (range, 10–46) in group 1 and 11 (range, 10–33) in group 2. (*P* = 0.17). The medium number of para-aortic lymph nodes removed was three (range, 0–23) in group1 and three (range, 0–11) in group 2 (*P* = 0.11). Among the cohort, 320 (36.61%) had undergone pelvic and para-aortic lymph node dissection and 530 (60.64%) had taken only pelvic lymphadenectomy. Meanwhile, 24 (2.75%) patients accepted para-aortic lymph node sampling. Univariate comparison between two groups was presented in Table 1.

**Table 1:** Clinical characteristics of stage I to III endometrial cancer patients stratified by lymph node status

Characteristics	Total *N* = 874	LNM		*P* value
		No *N* = 792 (90.62)	Yes *N* = 82 (9.38)	
Age at surgery (years)	53.58 ± 0.40	53.74 ± 0.42	51.79 ± 1.53	0.24
Pathological type				0.01
Endometrioid	760 (86.96)	702 (88.64)	58 (70.73)	
Non-endometroid	114 (13.04)	90 (11.36)	24 (29.27)	
FIGO stage				< 0.01
I	570 (65.22)	570 (71.97)	0	
II	212 (24.26)	212 (26.77)	0	
III	92 (10.52)	10 (1.26)	82 (100)	
Tumor grade				0.04
1	240 (27.46)	224 (28.28)	16 (19.51)	
2	438 (50.14)	398 (50.25)	34 (41.46)	
3	196 (22.40)	170 (21.57)	32 (39.03)	
Myometrial invasion				< 0.01
< 50%	586 (67.05)	548 (69.19)	38 (46.34)	
≥ 50%	288 (32.95)	244 (30.81)	44 (53.66)	
Primary tumor diameter				0.26
< 2	100 (11.44)	96 (12.12)	4 (4.88)	
≥ 2	774 (88.36)	696 (87.88)	78 (95.12)	
Cervical stromal invasion				< 0.01
No	604 (69.11)	576 (72.73)	28 (34.15)	
Yes	270 (30.89)	216 (27.17)	54 (65.85)	
LVSI				< 0.01
No	736 (84.21)	716 (90.40)	48 (58.54)	
Yes	138 (15.79)	76 (9.60)	34 (41.46)	
Ascites				0.49
No	736 (84.21)	670 (84.60)	66 (80.49)	
Yes	138 (15.79)	122 (15.40)	16 (19.51)	
Ovarian metastasis				< 0.01
No	850 (97.25)	782 (98.74)	68 (82.93)	
Yes	24 (2.75)	10 (1.26)	14 (17.07)	
Preoperative CA125	24.45 (27.70)	23.19 (26.52)	41.53 (56.46)	< 0.01
Lymphadenectomy				0.61
Only pelvic	530 (60.64)	478 (60.35)	52 (63.41)	
Pelvic + paraaortic	320 (36.61)	294 (37.12)	26 (31.71)	
Paraaortic sampling	24 (2.75)	20 (2.53)	4 (4.88)	
Median number of LNs removed, *n* (range)	13 (10–65)	13 (10–65)	13 (10–33)	0.83
Number of pelvic LNs removed	13 (10–46)	13 (10–46)	11 (10–33)	0.17
Number of paraoartic LNs removed	3 (0–23)	3 (0–23)	3 (0–11)	0.11
Lymph node metastasis				
Pelvic LN metastasis only	–	–	74 (90.24)	
PALN metastasis only			0	
Pelvic and PALN metastasis			8 (9.76)	
Status				< 0.01
Alive	790 (90.39)	748 (94.44)	42 (51.22)	
Died	84 (9.61)	44 (5.56)	40 (48.78)	
Follow-up, month (range)	59.3 (5–108.5)	59.5 (5–108.5)	56.6 (8.3–108)	0.96
Adjuvant therapy				0.03
No	748 (85.58)	690 (87.12)	58 (70.73)	
Chemotherapy only	94 (10.76)	74 (9.34)	20 (24.39)	
Radiotherapy only	28 (3.20)	24 (3.03)	4 (4.88)	
Both	4 (0.46)	4 (0.51)	0	

*LVSI* lymphovascular space invasion, *FIGO* international federation of gynecology and obstetrics. Values for continuous variables are mean ± standard deviation. Values for categorical variables are number (percentage). *LNs* lymph nodes, *PALN* para-aortic lymph node

A *P* value < 0.05 was considered to be statistically significant

The variables that showed statistical significance in univariate analysis (shown in Table 1) were included in a multivariate regression analysis. Multivariate comparison between two groups was shown in Table 2. No difference was noted in pathological type, tumor grade, deep myometrial invasion, and preoperative CA125 (see Table 2). Whereas cervical involvement, LVSI, and ovarian metastasis showed statistical significance (*P* < 0.05).

**Table 2:** Multivariate analysis of factors predictive of lymphatic dissemination using logistic regression models

Characteristics	Multivariate analysis	
	OR (95% CI)	*P* value
Pathological type		
Endometrioid	–	–
Non-endometrioid	2.107 (0.887–5.005)	0.09
Tumor grade		
1	–	–
2	1.140 (0.386–3.370)	0.81
3	1.065 (0.446–2.546)	0.89
Myometrial invasion	1.788 (0.830–3.851)	0.14
Cervical involvement	3.412 (1.631–7.141)	< 0.01
LVSI	2.542 (1.061–6.004)	0.04
Ovarian metastasis	6.236 (1.561–24.906)	0.01
Preoperative CA125	0.999 (0.997–1.001)	0.28

*OR* odds ratio, *CI* confidence interval, *LVSI* lymphovascular space invasion

A *P* value of < 0.05 was considered to be statistically significant

Conferent: pathological type (endometrioid); tumor grade (1); myometrial invasion (< 50%); cervical invasion (< 50%); LVSI (−); ovarian metastasis (−)

At the endpoint of the observation, a total of 790 (90.39%) patients survived, and 84 died for unknown reasons. The mean follow-up time was 59.3 months (range, 5–108.5) for all patients, 59.5 months (range, 5–108.5) in group 1, and 56.6 months (range, 8.3–108) for group 2 respectively. In univariate analysis, pathological type (*P* < 0.01), tumor grade (*P* < 0.01), myometrial invasion (*P* < 0.01), cervical involvement (*P* < 0.01), LVSI (*P* < 0.01), lymph node metastasis (*P* < 0.01), and ovarian metastasis (*P* < 0.01) were significantly associated with decreased overall survival. Regression model, as shown in Table 3, emphasized the pathological pattern (*P* = 0.03) and myometrial invasion (*P* = 0.02) as strong predictors of decreased overall survival (OS). In addition, lymph node metastasis (*P* < 0.01) and ovarian involvement (*P* = 0.01) were also confirmed as a potent sign of poor outcome.

**Table 3:** Multivariate analysis of overall survival in stage I–III endometrial cancer patients

Covariate	Overall survival		
	95% CI	Hazard ratio	*P* value
Pathological type			0.03
Endometrioid	–	–	
Non-endometrioid	1.133–6.839	2.784	
Myometrial invasion	1.139–6.400	2.700	0.02
Ovarian metastasis	1.603–50.477	8.994	0.01
Lymph node metastasis	3.708–25.245	9.675	< 0.01
Adjuvant therapy			0.18
No	–	–	0.06
Chemotherapy	0.857–329.210	16.792	0.03
Radiotherapy	1.376–760.366	32.350	0.15
Both	0.422–342.529	12.024	

*OR* odds ratio, *CI* confidence interval, *LVSI* lymphovascular space invasion

A *P* value of < 0.05 was considered to be statistically significant

Conferent: pathological type (endometrioid); myometrial invasion (< 50%); ovarian metastasis (−); lymph node metastasis (−); adjuvant therapy (no adjuvant therapy)

**Fig. 1:**
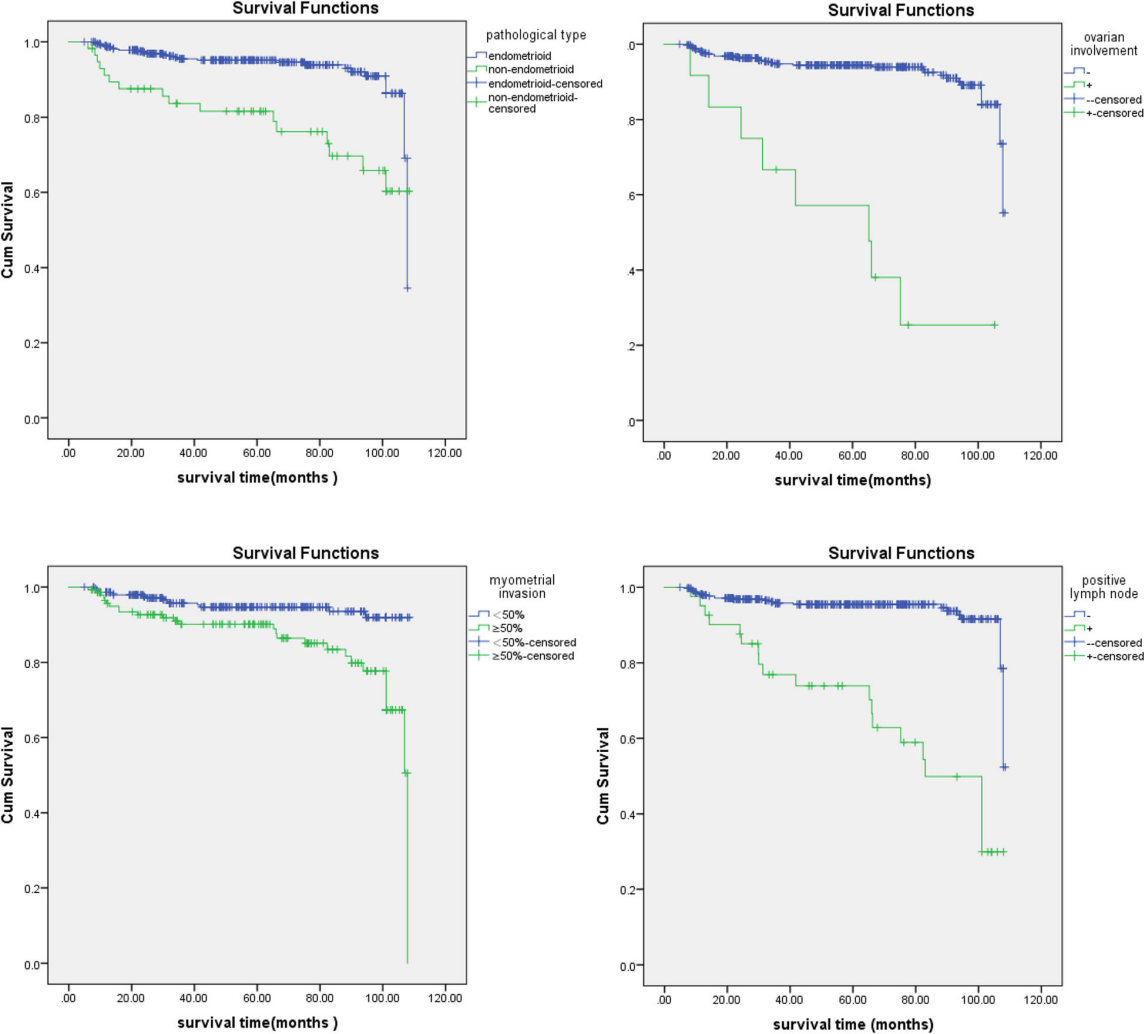
Kaplan–Meier curves for overall survival

**Fig. 2:**
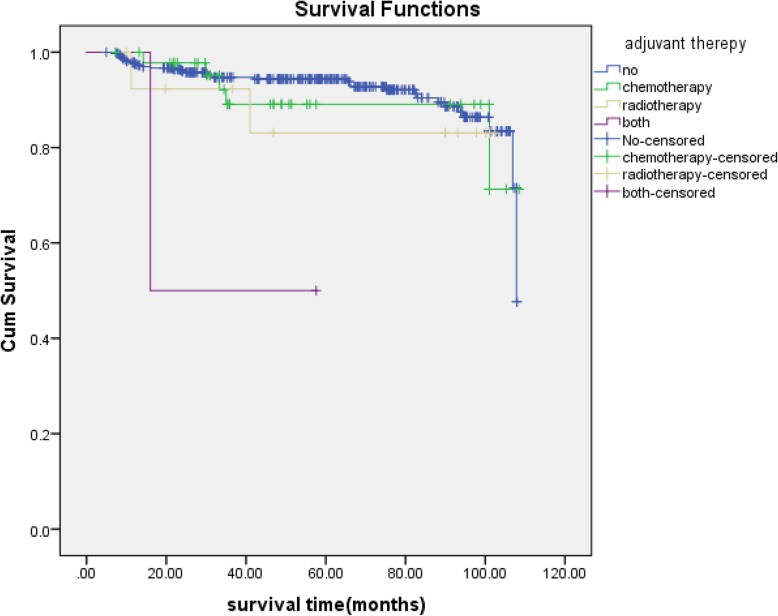
Overall survival according to adjuvant therepy

Cox curves for OS were plotted for the relationship of survival time with non-endometrioid type, deep myometrial invasion, ovarian metastasis, and positive lymph nodes (Fig. 1). Overall survival significantly decreased in patients with non-endometrioid type, deep myometrial invasion, ovarian and lymph nodes dissemination. (log rank test, *P* < 0.05). As for adjuvant therapy, Kaplan–Meier plot between two groups was shown in Fig. 2. And the log rank test revealed statistical significance(*P* = 0.04), whereas the multivariate analysis revealed no significance.

## Conclusions

In our study, we demonstrate that the following factors are observed to have separate significance for nodal metastasis: non-endometrioid histology, high tumor grade, deep (≥ 50%) myometrial invasion, cervical stromal involvement, lymphatic vascular invasion, ovarian involvement, and CA125. Meanwhile, we confirm that cervical stromal invasion, lymphopoiesis space invasion, and ovarian metastasis are independent predictors of nodal metastasis. Further, non-endometrioid carcinoma, deep myometrial invasion, ovarian metastasis, and lymph node metastasis are independent predictors of decreased survival. Different type of adjuvant therapy has undefined momentous influence on overall survival.

We find cervical invasion in 292 (29.80%) cases, which is in conformity with the range of cervical involvement in other literature [21]. The finding of a significant association between cervical involvement and LNM in this study is in concordance with previous studies [22] and stresses the importance of a thorough preoperative evaluation of cervical invasion. The two clinical studies by Cetinkaya et al. [23] and Boren et al. [22] investigated the relationship of cervical involvement with lymph node dissemination, and conveyed that positive cervical invasion was significantly associated with nodal metastasis in univariate analysis. However, it did not constitute an independent predictor, and led to a different result from our investigation. Previous studies showed that dynamic contrast-enhanced magnetic resonance imaging (CE-MRI) was the best tool to assess the cervical involvement [24]. The diagnostic accuracy of CE-MRI was 94.6%, which indicated pathological cervical invasion could be almost excluded in patients diagnosed by CE-MRI as not having cervical invasion [25]. However, the possible reasons for MRI inconsistency with final pathological findings are various [26]. More evidence is needed in order to prove this point.

A study by Akbayir et al. [13] in 2012 revealed that lymphopoiesis space invasion, cervical glandular, and stromal involvement were highly associated with lymph node involvement. Another paper proposed high-intermediate risk status and lymphatic vascular space infiltration were useful in assessing risk of pelvic nodal disease, and significantly reduced progression-free and overall survival [16]. More recently, Jorge et al. [27] identified 25,907 patients and confirmed lymphopoiesis space invasion was independently associated with lymph node dissemination and remained an independent predictor of survival. Consistent with the aforementioned researches, we also confirmed that lymphovascular space invasion was an independent predictor of lymph node dissemination.

So far, many researchers have undertaken to recognize the risk factors of nodal dissemination with endometrial carcinoma, and the conclusions are varied. In a nation-wide study, the Swedish gynecological cancer group [28] put forward that MI ≥ 50%, atypia histology, and FIGO grade 3 had a strong association with lymph node metastasis. A study by Kadan et al. [29] raised that lower body mass index was a danger factor of nodal metastasis in low-risk endometrial cancer. Wang et al. [8] proposed that CA125 could indicate whether a lymphadenectomy was required. Besides, Lucic et al. [30] considered tumor size > 2 cm, myometrial invasion ≥ 50%, and lymphopoiesis space invasion presented at G2 and G3 had high risk of lymphatic spread. Interestingly, all of the above parameters showed significance only in univariate analysis in this study. The cause of this difference may be the diverse inclusion criteria and deficiency of data. Therefore, additional studies are still essential.

In our findings, the absence of skip metastasis (para-aortic positive nodes only) is to some extent in accordance with results from previously published single-center investigation [31]. This may mean that pelvic lymph node dissection is possibly an acceptable alternative in selected patients, if surgery or patient-related conditions make it difficult to dissect the aortic artery. Minimally invasive surgery has developed rapidly and can improve the rehabilitation quality of women with endometrial cancer [32]. In our study, 16.5% patients received laparoscopy. The innovative technology even reduces the risk of surgery in older women, which may expand the scope of lymph node clearance [33]. At the same time, the introduction of sentinel lymph node mapping may further reduce surgical trauma. According to NCCN guideline, sentinel lymph node mapping can be regarded as the surgical stage of malignant tumors confined in uterine [7]. Nevertheless, there is no consensus regarding sentinel node mapping in clinical practice in ESGO-ESTRO-ESMO guidelines [34]. In our center, sentinel lymph node mapping is not extensively performed due to no consensus and technical limitations. Therefore, the data are not included in the study.

Poor outcome in endometrial cancer becomes increasingly important in clinical treatment. In our study, positive lymph node and ovarian involvement were related to poor outcome. Meanwhile, histology feature and deep myometrial invasion are strong predictors for diminishing survival. This is in line with Creutzberg et al. [35] Additionally, postoperative adjuvant therapy does have an effect on the overall survival, although without statistical significance, which is speculated as a result of its low percentage in the total subjects. This conclusion is inconsistent with the results of other contemporary studies [36]. In the PORTEC-1 and PORTEC-2 studies, the effect of EBRT and VRT on patients with high-intermediate risk was observed. Both studies concluded that neither EBRT nor VBT had a positive impact on overall survival compared with the subjects who did not receive adjuvant therapy [37, 38].

The limitations of the study lie in its retrospective nature, with which the accuracy of the results are negatively influenced by recall bias, selected bias, and confounding variables [9]. Moreover, during the 8-year research period, significant improvements in the clinical techniques also influence the result. In addition, some patients included in the study had incomplete data, which results in errors in the consequences and loss of true statistical significance. Despite these shortcomings, a large number of patients with specific clinical features were enrolled in this study, and the follow-up data available are reliable. Furthermore, as a single-center experience, all invasions treatments were performed by an experienced gynecologic oncologist.

In conclusion, cervical invasion, lymphopoiesis space invasion, and ovarian metastasis dramatically convey the risk of lymph node metastasis. Pathological type, myometrial invasion, and LVSI are all significant predictors of survival and should be completed when possible in the staging of patients with endometrial cancer. Further randomized controlled trials are needed to validate our results.

## Data Availability

The datasets used and/or analyzed during the current study are available from the corresponding author on reasonable request.

## Abbreviations

CE-MRI: Contrast-enhanced magnetic resonance imaging
CI: Confident interval
EC: Endometrial cancer
FIGO: International Federetion of Gynecology and Obstetrics
LNM: Lymph node metastasis
LVSI: Lymphovascular space invasion
NCCN: National Comprehensive Cancer Network
Od: Odd ratio
OS: Overall survival
PTD: Primary tumor size
